# Healthy Eating as Potential Mediator of Inverse Association between Purpose in Life and Waist Circumference: Emerging Evidence from US and Chilean Cohorts

**DOI:** 10.3390/ijerph20237099

**Published:** 2023-11-23

**Authors:** Loni Berkowitz, Camila Mateo, Cristian Salazar, Bárbara Samith, Daniela Sara, Victoria Pinto, Ximena Martínez, Mariana Calzada, Andrea von Schultzendorff, Nuria Pedrals, Marcela Bitran, Guadalupe Echeverría, Chiara Ruini, Carol Ryff, Attilio Rigotti

**Affiliations:** 1 Departamento de Nutrición, Diabetes y Metabolismo, Escuela de Medicina, Pontificia Universidad Católica, Santiago 8331150, Chile; loniberko@gmail.com (L.B.); bpsamith@uc.cl (B.S.); m.calzada@uc.cl (M.C.); npedrals@uc.cl (N.P.); gechevee@uc.cl (G.E.); 2 Centro de Nutrición Molecular y Enfermedades Crónicas, Escuela de Medicina, Pontificia Universidad Católica, Santiago 8331150, Chile; camateo@uc.cl (C.M.); cristian.salazar@uc.cl (C.S.); daniela.sara.zaror@gmail.com (D.S.); vspinto@uc.cl (V.P.); nut.ximenamartinez@gmail.com (X.M.); andreavonsch@gmail.com (A.v.S.); mbitran1609@gmail.com (M.B.); 3 Carrera de Nutrición y Dietética and Centro de Nutrición Molecular y Enfermedades Crónicas, Facultad de Medicina, Pontificia Universidad Católica, Santiago 8331150, Chile; 4 Department for Life Qualities Studies, University of Bologna, 40126 Rimini, Italy; chiara.ruini@unibo.it; 5 Institute on Aging, University of Wisconsin-Madison, Madison, WI 53715-1149, USA; cryff@wisc.edu

**Keywords:** purpose in life, healthy eating patterns, waist circumference, chronic diseases, cross-sectional studies, mediation analysis

## Abstract

High sense of purpose in life, a fundamental domain of eudaimonic well-being, has been consistently associated with lower risk for various obesity-related chronic diseases. Although this psychological feature correlates with some health behaviors as potential mediators, its association with healthy eating remains less explored. In addition, studies of these psycho-behavioral and health relationships in the South American population are lacking. This research sought to assess: (1) the cross-sectional association between self-reported purpose in life and overall healthy eating patterns, and (2) healthy food intake as a potential mediator of the inverse relationship between purpose in life and waist circumference. Data collected of 2060 US adults from the MIDUS study (5 ± 12 years, 55% women, mostly white people, and 42.5% obese) and 223 Chilean adults from the CHILEMED study (46.6 ± 9 years, 58.3% women, and 71.3% obese) were used. Anthropometric and sociodemographic variables were collected. Sense of purpose was assessed using the purpose in life subscale of the Ryff’s psychological well-being questionnaire. Diet quality was evaluated using healthy eating or low-fat diet indexes, according to extant food intake data in each cohort. The relationship between these variables was estimated by bivariate and multivariate linear regressions with appropriate adjustments. To establish whether a better diet quality could mediate a link of purpose in life and improved nutritional status (assessed by waist circumference), the association between these three variables was tested by bootstrapping-based mediation analysis. Our results show significant associations of sense of purpose with healthy eating and low-fat dietary patterns in both US and Chilean cohorts, respectively, even after adjusting for sociodemographic variables. According to the mediation analysis, the relationship between sense of purpose and waist circumference, as an indicator of abdominal obesity, appears to be partially mediated by healthier food intake in both samples. In conclusion, our findings suggest a plausible mechanism underlying the favorable impact of this well-being dimension on physical health. Given its protective effects, interventions aimed at increasing purpose in life may facilitate adherence to better dietary patterns, which, in turn, will reduce the risk for obesity-related chronic diseases.

## 1. Introduction

A strong sense of purpose in life has been proposed as an essential component of well-being [1], particularly with regard to the eudaimonic perspective of well-being [2]. This psychological feature has been conceptualized in various ways [1,2,3,4,5,6,7,8,9,10,11]. A sense of purpose in life has been defined as the extent to which an individual experiences their life as being directed and motivated by valued vital goals [2]. Another concept indicates that purpose in life is characterized by an overall and unwavering motivation and commitment to undertake valued life goals that are at once individually meaningful but also head to fruitful engagement with some aspects of life beyond the self [3]. This conceptual framework includes three key components: direction/goal orientation, personal sense/meaningfulness, and attention further than the self. It has also been associated with the Japanese term “ikigai”, which is defined as having the sense that one’s life is worth living [11].

Moreover, evidence shows that greater purpose of life and/or meaning in life, which have been used interchangeably, are positively related with better lifestyle behaviors [12], enhanced physiological functioning (e.g., less inflammation [13,14,15] and better glucoregulation [16,17,18,19]), and improved health outcomes, including reduced prevalence and/or incidence of diseases (e.g., cardiovascular disease (reviewed in [12,20]) and cognitive decline [21,22], as well as lower risk of mortality [20,23,24]). These findings are relevant due to the ongoing increase in non-communicable chronic diseases (NCDs) associated with poor habits and obesity [25], which are the leading causes of morbidity and mortality worldwide [26].

Building up observational and experimental studies have suggested that the potential benefits of a higher sense of purpose on physical health might may be explained by indirect effects through promotion of restorative and/or avoidance of harmful health behaviors (reviewed in [12]). Indeed, cross-sectional and longitudinal studies have found that a higher sense of purpose is associated with more physical activity [27,28,29], less smoking [30,31,32], better sleep [33], and increased use of preventive measures/resources [34]. 

Among modifiable lifestyle habits, an unhealthy diet is one of the most relevant risk factors for the development of NCDs [25]. Therefore, healthy nutrient and food intake can prevent or help to manage various NCDs. Despite the well-known benefits of healthy dietary patterns, their relationships with purpose in life have not been well evaluated. Indeed, many studies correlating this psychological feature with health behaviors have not considered diet or dietary components at all, even though a small number of studies have addressed this issue (reviewed in [12,35,36]). For instance, higher purpose in life was correlated prospectively with increased daily intake of vegetables or fruits and vegetables—as a proxy of healthy eating—in older adults from Hawaii [35] and the United Kingdom [36], respectively. As reported in these cohorts, studies have mostly evaluated intakes of specific food items (e.g., vegetables and/or fruits) with regard to life purpose, rather than using more comprehensive scores of healthy eating patterns. These latter are sounder measures of overall real-life food intake, more appropriately detect dietary changes in time, and correlate/predict better various clinical outcomes [37,38]. Thus, to date, there are no studies specifically appraising the relationship between purpose in life and overall dietary patterns. Furthermore, it is unknown whether this relationship could mediate the positive association between sense of purpose and a variety of physical and mental health outcomes mentioned above. If so, interventions aimed at increasing purpose in life may facilitate adherence to better dietary patterns, which, in turn, will reduce the risk for NCDs.

When studying purpose, food intake, and their interrelationships, it is essential to consider significant cultural differences as they can help to contextualize the observed patterns and contribute to a better understanding of how eating behaviors and senses of purpose vary and interact across different national backgrounds [39,40]. The reasons for these differences are multifaceted and can be attributed to historical, social, economic, and environmental factors that can impact cultural norms and behaviors related to food consumption and purpose in life.

Given the importance of the high prevalence of NCDs derived from obesity and poor dietary habits, it is essential to further evaluate whether a higher sense of purpose is associated with better dietary habits and obesity-related anthropometrics. Using US and Chilean cohorts, the aim of this study was to assess: (1) the cross-sectional association between self-reported purpose in life and overall healthy eating patterns, and (2) healthy food intake as a potential mediator of the inverse relationship between purpose in life and waist circumference, a well-known parameter to assess abdominal obesity. 

## 2. Materials and Methods

### 2.1. Samples

Data for the current analysis were collected as part of two ongoing studies: one from the United States (MIDUS) and another from Chile (CHILEMED). 

The MIDUS (Midlife in the United States) study was initiated in 1995 to assess the impact of psychological, behavioral, and social factors in adult health (MIDUS 1), mostly in a national representative non-institutionalized random digit dialing-selected sample. MIDUS 2 was a longitudinal follow-up conducted 9–10 years after the initial survey and involved a thorough examination of biomarkers in a select MIDUS group. Every attempt was made to contact all original respondents and invite them to participate in a second wave of data collection. To increase the representation of age and racial diversity, MIDUS Refresher recruited a fresh set of participants following the 2007–2009 great recession. From subjects of MIDUS 2 and MIDUS Refresher who participated in the survey and the biomarker project (n = 2118), we removed 58 individuals (2.7%) who did not have psychological or nutritional measures. Thus, the current analysis included cross-sectional data of 2060 subjects. Detailed information regarding the sample compositions is available elsewhere [41].

CHILEMED is an interventional study (Clinical Trials NCT05454904) to be carried out between 2021 and 2024 and aims to evaluate the impact of healthy dietary patterns and a psychological wellbeing-based intervention on the reversal of metabolic syndrome. Metabolic syndrome was diagnosed with the following guidelines from Ministerio de Salud (MINSAL) of Chile [42] based on the US National Cholesterol Education Program (NCEP) [43]. More information regarding the inclusion and exclusion criteria is available elsewhere [44]. This cohort is expected to include more than 330 Chilean participants 25 to 70 years-old with metabolic syndrome, providing an appropriate setting to conduct nested studies to investigate variables associated with cardiometabolic health. For this analysis, baseline data collected up to March 2023 (n = 223) were used from patients who had undergone nutritional evaluation and responded to psychological questionnaires. 

### 2.2. Purpose in Life Assessment 

Sense of purpose was assessed using the purpose in life subscale of the Ryff’s psychological well-being questionnaire. This questionnaire evaluates a general/overall sense of purpose and direction in life based on personal goals (2), but it does not include additional elements (e.g., beyond the self aims and additional sources of meaning) that have been considered in other conceptual frameworks (3–11). In MIDUS, respondents indicated their agreement with seven items on a 7-point Likert-type scale [2]. In CHILEMED, the Spanish version of the Ryff’s questionnaire was used [45], whose purpose in life subscale has 6 items. In both cases, scorings for each single item were summed up to obtain an overall measure of sense of purpose, in which higher scores indicated higher purpose in life.

### 2.3. Healthy Eating Pattern Evaluation

Dietary quality was assessed using data from self-applicable questionnaires and categorized using validated indexes, according to the information available in each cohort. 

To assess quality of food consumption in MIDUS, a healthy eating index was used (MIDUS-HEI), which categorized respondents from 0 to 11 points by evaluating intake of sugary beverages, vegetables, fruits, whole grains, fish, fat meat, lean meat, non-meat protein, fast food, alcohol, and fermented dairy products [Echeverría et al., under revision]. In CHILEMED, a 0- to 10-points score of compliance with a low-fat dietary pattern (low-fat diet index, LFDI, modified from [46]) was used evaluating food intake as follows: vegetable oil, high-fat meats (e.g., hamburgers, sausage, bacon, and salami), visible fat removal from meats, low fat vs. high fat dairy products, oily fish, ultra-processed fatty foods, fried food, nuts, and avocado. In both indexes, higher scores indicated high adherence to a healthier diet.

### 2.4. Sociodemographic, Anthropometric, and Physical Activity Information 

A number of demographic and health variables were measured in both cohorts. Self-reported sociodemographic factors included sex (i.e., men or women), race (i.e., white or non-white), age as numeric (years-old) or categoric variables (<50, 50–65, and >65 years), and educational level (i.e., high school or less, college education or equivalent, and postgraduate studies). Physical anthropometrics, including body mass index (BMI, kg/m^2^) and waist circumference (cm), were measured by the staff of the corresponding clinical research centers. For the description of nutritional status, BMI was classified into underweight or normal weight (BMI < 25 kg/m^2^), overweight (25 kg/m^2^ ≤ BMI < 30 kg/m^2^), or obese (BMI ≥ 30 kg/m^2^). Physical activity was self-reported and subsequently transformed into metabolic equivalents for task per week and categorized (>450 METs/week and ≤450 METs/week).

### 2.5. Statistics

Participant characteristics were summarized as the mean ± SD for continuous variables or percent occurrences for categorical variables. Statistical analyses were conducted using R-studios Desktop, version 1.2.5001. All analyses were two-tailed and considered statistically significant differences when *p* ≤ 0.05.

Student’s *t* or one-way ANOVA tests were used to compare healthy eating scores (i.e., MIDUS-HEI or CHILEMED-LFDI) and sense of purpose by sociodemographic variables and nutritional status in both cohorts. Associations between healthy eating indexes and purpose in life were evaluated by bivariate or multivariate linear regression models (adjusting for sex, age, race, and education attainment) with normalized data using z-scoring. 

To investigate whether healthy eating was a potential mediator of the relationship between sense of purpose and waist circumference as a marker of abdominal obesity, percentile bootstrapping-based mediation analysis was performed [47]. If 0 landed outside of the confidence interval (CI), mediation was likely, whereas there was no statistical evidence for mediation if 0 was within the CI. Mean indirect and total effects were computed across 5000 bootstrap samples to represent the final indirect effect estimate. For this analysis, waist circumference values were log-transformed to achieve normal data distribution, whereas sex, age, race, education, and physical activity were used as covariables.

### 2.6. Ethics

The MIDUS study protocol was approved by the Institutional Review Board at the University of Wisconsin–Madison, University of California, Los Angeles, and Georgetown University. CHILEMED study was approved by the Ethics Committee on Human Research at Pontificia Universidad Católica de Chile. Both studies were conducted in accordance with the Declaration of Helsinki, and participants provided written informed consent before screening and recruitment. All data were handled anonymously.

## 3. Results

### 3.1. Healthy Eating Indexes and Sense of Purpose by Sociodemographic Characteristics and Nutritional Status in MIDUS and CHILEMED 

The MIDUS sample comprised 2060 middle-aged and older adults (mean 55.6 ± 12 years-old) from the US: over half were women, mostly white people; three quarters had a higher education (i.e., college or postgraduate studies); about one quarter of them showed normal weight; and 61% reported physical activity >450 METs/week (Table 1). On the other hand, the CHILEMED sample encompassed 223 middle-aged Chilean adults, most of them under 65 years old (mean 46.6 ± 9 years-old), over half of them were women, and more than three quarters of the sample had at least a college education (Table 1). Unlike the MIDUS sample, all participants in CHILEMED were Hispanic people with metabolic syndrome, and less than 5% of the sample had normal body weight, exhibited increased waist circumference, and reported lower physical activity.

Regarding the food intake pattern—assessed by a healthy eating index (HEI) in subjects from MIDUS—a better score was observed in women versus men, in older age groups, in white subjects, and in individuals with higher educational levels and reporting more physical activity (Table 2). When analyzing different nutritional status groups, a significantly lower MIDUS-HEI score was found in overweight and obese subjects. Similar patterns were observed for the sense of purpose, with higher purpose in life scoring in women than men, in whites than non-whites, in older groups, in individuals with higher educational levels, and in those with better nutritional statuses and more physical activity (Table 2).

Overall, the LFDI and sense of purpose evaluated in CHILEMED showed similar trends to those detected in MIDUS with regard to sociodemographic variables, even though most comparisons did not reach statistically significant differences. Particularly, the CHILEMED-LFDI was significantly higher in older age groups (Table 2). Surprisingly, the lower education group of CHILEMED had a higher sense of purpose in life (Table 2). Physical activity did not correlate with LFDI or purpose in life.

### 3.2. Association between Sense of Purpose and Healthy Eating in MIDUS and CHILEMED

To further examine the relationship between sense of purpose and healthy eating, hierarchical regression analyses were conducted in both study samples (Table 3). 

First, the bivariate model showed that sense of purpose significantly correlated in a positive way with the healthy eating index in MIDUS. In model 2, after adjusting for age, sex, and race, the magnitude of the association between sense of purpose and MIDUS-HEI decreased, but it persisted as strongly significant. Something similar happened in model 3 after adjustments by educational level. Although all covariates showed significant effects, the positive association between purpose in life and healthy eating in MIDUS remained statistically substantial (Table 3).

When performing hierarchical regression analysis using CHILEMED data, comparable results were obtained (Table 3). The bivariate model displayed that higher sense of purpose significantly correlated with LFDI as an indicator of a healthier eating pattern. This association remained significant after adjusting for sex and age (model 2) and even after adding the educational level (model 3). Of these covariates, age and educational level, but not sex, showed a significant association with the low-fat dietary pattern.

### 3.3. Inverse Relationship between Purpose in Life and Waist Circumference: Healthy Eating as Potential Mediator 

Considering the significant association between a higher sense of purpose and healthier eating patterns in both cohorts, we evaluated whether this psychological feature and lifestyle behavior could also be associated with better nutritional status. For this, waist circumference was applied as an indicator of visceral fat accumulation and abdominal obesity, a well-known risk factor for various NCDs. Sex, age, race, and education attainment were used as covariables.

In both cohorts, significant direct effects were found for healthy eating (measured as HEI in MIDUS or LFDI in CHILEMED) on waist circumference and for sense of purpose on healthy eating (Table 4). The direct effect of purpose in life on waist circumference (adjusted for healthy eating) was statistically significant only in MIDUS (Table 4).

**Figure 1:**
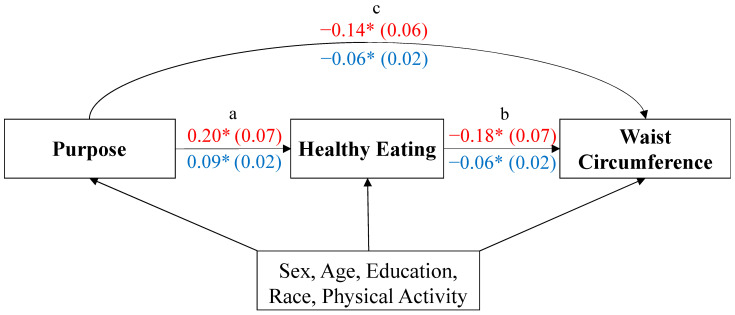
Mediation model of healthy eating on the associations between purpose in life and waist circumference in MIDUS and CHILEMED. Standardized regression coefficients and standard errors (in parentheses) are shown. The asterisk indicates *p* < 0.05. MIDUS effects are denoted in red, whereas CHILEMED effects are shown in blue. (a) Effect of purpose on healthy eating. (b) Effect of healthy eating (HEI or LFDI) on waist circumference. (c) Total effect of purpose on waist circumference.

A mediation bootstrapping analysis was conducted to statistically assess the mediating effect of healthy diet indexes on the relationship between higher sense of purpose and smaller waist circumference (Table 5). Based on this analysis, the indirect effect—mediated by a healthy eating pattern—was significant in both MIDUS and CHILEMED cohorts, as well as the total effect of purpose in life on waist circumference (Table 4 and Figure 1).

## 4. Discussion

Previous research (reviewed in [12,48,49]) indicated a positive relationship between healthy lifestyle behaviors (e.g., fruits and/or vegetable intake, adequate sleep habit, greater physical activity, moderate alcohol consumption, and not smoking), various positive psychological resources (e.g., optimism, vitality, and hope), and well-being components (e.g., positive emotions, happiness, and life satisfaction), thus promoting good health and reducing the risk of NCDs. In this study, we aimed to evaluate whether healthy eating was a potential mediator of an inverse association between purpose in life and waist circumference, a surrogate anthropometric marker of abdominal obesity. Based in two independent cohorts, one from the US and another from Chile, we found that cross-sectional purpose in life was positively associated by bivariate and multivariate linear regressions with overall healthy eating patterns, and these later appeared to significantly mediate—based on bootstrapping analysis—the inverse relationship between purpose in life and waist circumference.

Purpose in life is a crucial component of well-being and has been linked to numerous physical and health benefits [12,13,14,15,16,17,18,19,20,21,22,23,24]. These findings are correlational in nature but do not establish a cause–effect relationship. However, a sense of purpose in life may promote health and reduced disease risk due to different underlying plausible biological, psychological, stress-buffering, and behavioral mechanisms (reviewed in [12,27,28,29,30,31,32,33,34,35,36]). On the specific issue of food habits, however, fewer studies have investigated the relationship between healthy eating and purpose in life (reviewed in [12,35,36]). In addition, these studies handled limited evaluation of food intake, focusing on some specific food items (e.g., fruits and/or vegetables [35,36]), rather than using more comprehensive eating pattern scores. 

Our findings further support and extend this limited evidence by adding new information on the relationship between purpose in life and healthy eating. Importantly, we found a significant correlation—regardless of sex, age, and education level—between this psychological measure of well-being with two different and more integrative and representative scores to assess an overall salutogenic food intake, such as a healthy eating index in MIDUS and a low-fat diet score in CHILEMED. Results reinforce and widen the previously described association with life purpose from some selected beneficial food items towards overall healthy eating patterns. Interestingly, similar results have been reported when linking ikigai with diversity [50] or healthful [51] food intake scores in Japanese cohorts, demonstrating data consistency for these associations in Eastern and Western populations.

By applying mediation analysis, our findings indicate that a high sense of purpose in life links inversely with waist circumference through healthy eating patterns. This is fully consistent with the proposal that one mechanism by which life purpose may lead to better health and reduced risk of NCD conditions—in this case, abdominal obesity—is mediated indirectly through protective/restorative behaviors, including healthy eating [12]. People who have a strong sense of purpose may be prone to make healthier food choices and adhere to healthy eating patterns more consistently than those who lack a sense of purpose. This is likely because individuals who have a clear sense of purpose are more motivated to take care of their health and well-being, which includes eating a healthy and balanced diet. On the other hand, purpose in life can be increased by positive psychological interventions [52,53,54,55]. If so, promoting and increasing life purpose may have a significant effect in grounding and maintaining healthy eating with impact on reducing incidence and improving treatment of NCDs.

However, our cross-sectional analysis does not rule out reverse causality, and the mechanistic link between healthy eating and purpose in life could be bidirectional. In fact, purpose in life may be an outcome rather than a primary determinant of its relationship with health-related lifestyle behaviors. For instance, some studies have suggested that engaging in a healthier lifestyle also predicts a greater purpose in life [51]. In this regard, additional longitudinal analyses should help to address this issue. If so, a diet that is rich in fruits, vegetables, whole grains, legumes, oily fish, and lean proteins provides the nutrients necessary for optimal brain function, which can enhance cognitive function, mood, and overall well-being. Eating a healthy diet can also promote physical health, which enables individuals to pursue their goals and find meaning in life. For example, subjects who are physically healthy may be able to participate in activities that they enjoy, pursue their career goals, or spend quality time with loved ones, all of which can contribute to a greater sense of purpose. 

As mentioned above, cultural differences between populations, such the US and Chilean cohorts used in this study, should be considered when contextualizing the major findings and understanding how eating behaviors, diet quality, and sense of purpose vary and interact in different cultural frameworks [39,40]. For instance, as a consequence of convenience, availability, and socioeconomic factors, the US is often characterized by an overarching trend to a more Westernized and less healthy diet with high intake of processed foods, sugary beverages, and fast food, which contribute to obesity and related NCDs [56]. In contrast, Chilean traditions exhibit healthier food patterns and cuisine using a variety of fruits, vegetables, grains, and seafood, mimicking a Mediterranean pattern, which reflects cultural heritage and local produce availability [57,58,59]. However, like many other Western countries, Chile is also experiencing a nutritional transition towards increased consumption of processed and high-calorie foods [60].

Cultural values and norms also play significant roles in shaping individuals’ senses of purpose. The US culture often places an emphasis on independence, individualism, self-determination, and personal achievement as key components of one’s life purpose and goals [61]. In contrast, Chilean culture tends to be more interdependent and collectivist, with greater emphasis on family, community, and social connections, influencing conceptualization and experience of purpose [62].

Furthermore, the interrelationships between eating patterns and senses of purpose can be influenced by cultural norms and contexts. For instance, in individualistic cultures like the US, personal achievement might be linked to certain dietary choices, such as following fad diets for improving physical appearance and body image. In collectivist populations like Chile, eating patterns may be more affected by communal values and social gatherings, leading to shared meals and traditional foods being central to family and group identity. Cultural values related to health and well-being can also affect individuals’ purposes and their approaches to food choices. For example, a person in US might find purpose in adhering to a health-oriented diet, whereas a subject in Chile could find more purpose in preserving cultural food traditions.

Strengths of the study. First, we used compound food intake scores instead of single food groups to assess diet quality. This is relevant because the focus in nutritional epidemiology is shifting from specific food components to overall eating patterns [37]. Indeed, analyses of individual foods, rather than global dietary patterns, have shown to be more susceptible to misreporting [38]. Nutritional research on dietary patterns is also more understandable and adoptable for people and easier to be translated into public health recommendations and implementations, even before the mechanisms determining the observed associations are well defined. Finally, using culturally distinctive cohorts as well as two different diet scores provide independent and external data validity, generating reproducibility and consistency in the associations, regardless of the measures applied and the specific populations assessed. Indeed, our findings in the Chilean cohort further extend the evidence previously reported in the US, European, and Japanese samples [35,36,50,51].

Limitations of the study. First, dietary assessments were self-reported, which may cause bias, thus negatively impacting validity of the findings. Under-reporting and misreporting of dietary components in self-reported data are well-known, resulting in spurious diet–disease associations [63]. Future studies may better assess food intake by using more detailed (e.g., 24 h food recalls) and/or more objective and robust (e.g., dietary metabolomic signature) approaches. Second, a smaller sample size in the CHILEMED cohort may have limited the statistical power to reproduce extensive evidence showing consistent relationships between age and sex with healthy eating patterns, as they occurred in MIDUS. Indeed, comparing cohorts with significantly different sample sizes may cause issues. Smaller sample sizes may lack representativeness and statistical power, leading to less precise estimates and impaired detection of small effects. Additionally, selection biases and outliers can disproportionately impact studies with small sample sizes. Third, the cross-sectional design did not allow us to infer cause–effect relationships. As mentioned above, the directional causal link between purpose in life and better diet quality should be further investigated prospectively and, eventually, addressed in interventional trials.

## 5. Conclusions

In the US and Chilean cohorts, we found a strong relationship between purpose in life and healthy eating patterns, which, in turn, are associated with reduced waist circumference. Our findings suggest a plausible mechanism (i.e., better diet behavior) underlying the favorable impact of purpose in life, a key eudaimonic well-being dimension, on physical health. Given its potential protective effects on abdominal obesity, interventions aimed at increasing purpose in life in clinical and non-clinical subjects and/or contexts may facilitate adherence to better dietary patterns, which, in turn, will reduce the risk for obesity-related chronic diseases.

## Figures and Tables

**Table 1:** Sociodemographic features, nutritional status, and physical activity in MIDUS and CHILEMED.

	Study Samples	
	MIDUS (n = 2060)	CHILEMED (n = 223)
Sex, % (n)		
Men	45.3 (934)	41.7 (93)
Women	54.7 (1126)	58.3 (130)
Age, % (n)		
<50 years	33.6 (692)	64.6 (144)
50–65 years	43.3 (891)	31.8 (71)
>65 years	23.1 (477)	3.6 (8)
Race % (n)		
White (Caucasians)	74.9 (1543)	-
Non-White	25.1 (517)	-
Hispanic	-	100 (223)
Educational level, % (n)		
High school or less	23.7 (488)	23.3 (52)
College education	51.9 (1070)	56.1 (125)
Postgraduate studies	24.4 (502)	20.6 (46)
Nutritional status, % (n)		
Under or normal weight	23.9 (491)	4.5 (10)
Overweight	33.1 (681)	24.2 (54)
Obesity	42.5 (876)	71.3 (159)
Waist circumference, cm ± SD	98.0 ± 17.9	104.9 ± 9.7
Physical activity, % (n)		
>450 METs/week	60.8 (1252)	35.0 (78)
≤450 METs/week	39.2 (808)	65.0 (145)

**Table 2:** Healthy eating index and sense of purpose by sociodemographic features, nutritional status, and physical activity in MIDUS and CHILEMED.

	MIDUS Sample				CHILEMED Sample			
	HEI (Mean Score)	*p*-Value	Purpose in Life (Mean Score)	*p*-Value	LFDI (Mean Score)	*p*-Value	Purpose in Life (Mean Score)	*p*-Value
Sex								
Men	5.79	<0.001	38.74	0.004	6.50	0.45	29.3	0.48
Women	6.06		39.62		6.65		28.8	
Age								
<50 years	5.66	<0.001	38.83	<0.001	6.40	0.01	28.40	0.06
50–65 years	5.96		39.34		6.85		30.06	
>65 years	6.32		39.58		7.63		31.38	
Race								
White	6.10	<0.001	39.30	0.41				
Non-White	5.46		38.99					
Educational level								
Higher education or less	5.40	<0.001	37.79	<0.001	6.39	0.36	30.08	0.02
College education	5.96		39.22		6.71		28.84	
Postgraduate studies	6.43		40.61		6.50		28.37	
Nutritional Status								
Under or normal weight	6.12	<0.001	39.59	0.02	6.90	0.14	30.70	0.22
Overweight	5.98		39.65		6.88		29.91	
Obesity	5.81		38.69		6.47		28.63	
Physical activity								
>450 METs/week	6.13	<0.001	39.93	<0.001	6.61	0.87	28.29	0.14
≤450 METs/week	5.63		38.12		6.58		29.43	

**Table 3:** Sociodemographic-based multivariate association analysis of the relationship between healthy eating and purpose in life in MIDUS and CHILEMED.

	Model 1		Model 2		Model 3	
	Coef	*p*-Value	Coef	*p*-Value	Coef	*p*-Value
**MIDUS HEI**						
**PURPOSE IN LIFE**	0.153	<0.001	0.137	<0.001	0.108	<0.001
Sex			0.211	<0.001	0.237	<0.001
Age			0.124	<0.001	0.134	<0.001
Race			−0.38	<0.001	−0.305	<0.001
Education					0.199	<0.001
**CHILEMED LFDI**						
**PURPOSE IN LIFE**	0.208	0.002	0.151	0.022	0.163	0.014
Sex			0.096	0.458	0.161	0.224
Age			0.259	<0.001	0.270	<0.001
Education					0.365	0.024

Model 1: bivariate analysis (Model 1) between purpose in life and healthy eating (HEI and LDFI in MIDUS and CHILEMED, respectively). Model 2: adjusted for sex, age, and race. Model 3: adding education level.

**Table 4:** Associations between purpose in life, healthy eating, and waist circumference in MIDUS and CHILEMED.

Sample	Independent Variable	Dependent Variable	Coef	SE	*p*-Value
MIDUS					
	Purpose in life †	Waist circumference	−0.075	0.021	<0.001
	HEI	Waist circumference	−0.078	0.022	<0.001
	Purpose in life	HEI	0.108	0.021	<0.001
CHILEMED					
	Purpose in life †	Waist circumference	−0.104	0.065	0.107
	LFDI	Waist circumference	−0.18	0.064	0.005
	Purpose in life	LFDI	0.191	0.064	0.003

† Direct association between purpose and waist circumference adjusted for healthy eating (HEI or LFDI, respectively) in MIDUS and CHILEMED.

**Table 5:** Bootstrapping analysis of indirect and total effects of purpose in life on waist circumference mediated by healthy eating in MIDUS and CHILEMED.

Sample	Pathway	Effect	Coef	SE	95% CI
MIDUS					
	Purpose → Waist	Direct	−0.075	0.022	−0.12, −0.029
	Purpose → HEI → Waist	Indirect	−0.008	0.003	−0.015, −0.003
	Purpose → → Waist	Total	−0.083	0.022	−0.127, −0.037
CHILEMED					
	Purpose → Waist	Direct	−0.104	0.061	−0.215, 0.024
	Purpose → LFDI → Waist	Indirect	−0.034	0.018	−0.076, −0.005
	Purpose → → Waist	Total	−0.139	0.06	−0.247, −0.011

HEI: Healthy eating index in MIDUS; LFDI: Low-fat dietary index in CHILEMED.

## Data Availability

The data from the MIDUS cohort is publicly available and can be found here: https://midus.colectica.org/ (accessed on 1 May 2023). The data from CHILEMED that support the findings of this study are available from the corresponding author (A.R.) upon reasonable request.
